# Differential attraction in mosquito–human interactions and implications for disease control

**DOI:** 10.1098/rstb.2019.0811

**Published:** 2020-12-28

**Authors:** Julien Martinez, Alicia Showering, Catherine Oke, Robert T. Jones, James G. Logan

**Affiliations:** Department of Disease Control, Faculty of Infectious and Tropical Diseases, London School of Hygiene and Tropical Medicine, Keppel Street, London WC1E 7HT, UK

**Keywords:** attractiveness to mosquitoes, human host, skin microbiome, malaria parasites

## Abstract

Mosquito-borne diseases are a major burden on human health worldwide and their eradication through vector control methods remains challenging. In particular, the success of vector control interventions for targeting diseases such as malaria is under threat, in part due to the evolution of insecticide resistance, while for other diseases effective control solutions are still lacking. The rate at which mosquitoes encounter and bite humans is a key determinant of their capacity for disease transmission. Future progress is strongly reliant on improving our understanding of the mechanisms leading to a mosquito bite. Here, we review the biological factors known to influence the attractiveness of mosquitoes to humans, such as body odour, the skin microbiome, genetics and infection by parasites. We identify the knowledge gaps around the relative contribution of each factor, and the potential links between them, as well as the role of natural selection in shaping vector–host–parasite interactions. Finally, we argue that addressing these questions will contribute to improving current tools and the development of novel interventions for the future.

This article is part of the theme issue ‘Novel control strategies for mosquito-borne diseases'.

## 1. Introduction

Mosquito-borne diseases are a major cause of morbidity and mortality in human populations living in tropical and sub-tropical regions. A striking example is malaria, a disease transmitted by *Anopheles* mosquitoes that causes more than 400 000 deaths each year [1]. Despite the past and current successes of malaria control worldwide, progress in the eradication of the disease has stalled, in part owing to suboptimal intervention coverage and funding constraints, suggesting that global eradication is still a long way off [1]. Although there are recent advances in the development of vaccines against malaria and other mosquito-borne diseases such as dengue [2], vector control remains the main method of disease prevention. The most common tools are long-lasting insecticide-treated bed nets (LLINs) and indoor residual insecticide spraying (IRS) [3]. While vector management has proved to be one of the most effective ways to reduce disease transmission, control methods that are successful today may soon lose their efficacy owing to rapidly evolving mosquito populations [4]. The spread of resistance to insecticides is a concern in many parts of the world [5–7], especially pyrethroid resistance as pyrethroids are the major class of insecticides used in WHO-recommended LLINs [8]. Evidence of behavioural changes in mosquito feeding in response to LLINs and IRS, termed ‘behavioural resistance’, has also been reported [9], with several examples of mosquito populations becoming exophagic (i.e. outdoor biting) following the introduction of LLINs or IRS; however, measuring such changes remains challenging [10,11]. Future progress will require the development of innovative tools to protect human populations, and this can only be achieved once the complex biology behind vector–host interactions is understood better.

The transmission of mosquito-borne diseases requires direct contact between the vector and host when a blood meal is taken. Consequently, the vector–host contact rate is a key parameter of the parasite or pathogen's epidemiology as it is directly linked to its basic reproduction number (*R*_0_), a key measure of transmissibility [12]. The contact rate between humans and mosquitoes varies with the local abundance of vectors, vector host preferences and host attractiveness, which drive the likelihood of mosquito bites [13,14]. Many studies have demonstrated that some people attract more mosquitoes than others in laboratory studies [14,15], and interestingly, strong heterogeneities in exposure to mosquito bites have been observed at a local scale in the field, whereby a small fraction of people tend to receive most of the bites within a household [16]. Attractiveness has been shown to be mediated by differences in body odour [14], but the underlying biological factors are less well understood. Unravelling this is important because these heterogeneities are predicted to have a profound impact on the fraction of hosts and vectors carrying the parasite and the incidence of severe disease [12]. In populations where the *R*_0_ is high, targeting transmission to those that are bitten the most could help disease control [12]. Finally, while there is evidence that differences in host attractiveness and mosquito behaviour have some genetic basis, the roles of natural selection and coevolution between the interacting partners remain poorly understood.

Here, we review the biological factors that influence the contact rate between mosquitoes and humans, and subsequently the risk of exposure to deadly diseases. We highlight the potential role of individual variation, in both human attractiveness and mosquito feeding behaviour, in driving heterogeneities of biting frequency. We also explore the importance of genetic variation and how it may fuel natural selection acting on vector, human and parasite populations. We describe how taking into account individual variation will help improve the predictive power of epidemiological models. Finally, we discuss how a better understanding of host–parasite interaction could lead to the development of novel or improved control methods.

## 2. Mosquito–human interactions in a nutshell

**Figure 1.**
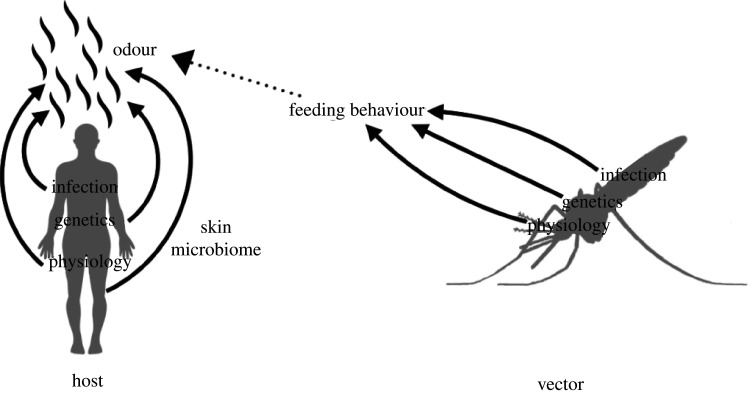
Potential factors affecting human attractiveness and mosquito feeding behaviour.

The contact rate between mosquitoes and their hosts is the outcome of a complex sequence of mosquito behaviours, including flight activation, attraction, landing and probing [17]. If accomplished this sequence can allow the transmission of infectious agents, including viruses and parasites, between the two organisms. Each of these behaviours is under the influence of both vector and host biological traits. Female mosquitoes have an innate motivation to locate and feed on certain blood hosts, and human hosts emit signals that either attract or repel mosquitoes. While much progress has been made to decipher how vectors locate their hosts and what makes humans attractive to them, there is still much to learn about the biological factors underlying individual variation in the two organisms. In particular, it is known that vectors can vary in their host preferences and that levels of attractiveness differ among human hosts [14,18]. Both genetic and non-genetic factors have been invoked to explain this variation, but their relative contribution and potential interaction remain poorly understood (figure 1). While abiotic environmental factors such as temperature or humidity are also important, we will only discuss the influence of biotic factors for the purpose of this review.

### (a) Mosquito feeding behaviour: a matter of taste

Host-seeking behaviour is activated over a long range (55–70 m) in the presence of carbon dioxide (CO_2_) exhaled from the mouth or released through the skin of the host [19–21]. As mosquitoes fly towards the host, they also detect other signals, such as visual cues, humidity and temperature gradients [22]. At close range, other volatile organic compounds (VOCs) released from the body of the host play an important role, in synergy with CO_2_, informing the mosquito of a potential blood source. Mosquitoes then land on their host and start probing the skin to take a blood meal. Olfaction is a major component of mosquito feeding behaviour and is known to be governed by numerous chemosensory genes such as those encoding olfactory receptors (ORs), odorant-binding proteins (OBPs) and ionotropic receptors (IRs) [23,24].

Mosquitoes show extensive variation in their host preference, in particular when it comes to choice between different host species [18,25,26]. Some mosquito species are defined as zoophilic as they feed on birds or mammals, while others are anthropophilic, meaning they show a strong preference for humans [18]. For instance, among *Anopheles* mosquitoes, 30 species out of more than 400 have been found to feed on humans [25]. Some *Anopheles* species, including *Anopheles gambiae* sensu stricto, *Anopheles coluzzii*, *Anopheles funestus* and *Anopheles stephensi*, are strongly attracted to human hosts and are significant vectors of human malaria parasites [25]. Other species, such as *Anopheles arabiensis*, lie at intermediate positions along the zoophily–anthropophily continuum and are considered opportunistic blood feeders. Such opportunism is expected in the context of heterogeneous spatial distribution of hosts since seeking a suitable host is time-consuming and costly in energy. There is evidence that mosquitoes can learn and adapt their behaviour based on prior experience [18,27], and demonstrate behavioural plasticity when their preferred host species is unavailable [28]. Mosquito species are thought to maximize their reproductive success by tuning their preference based on the availability of a particular host species [28–30].

There is strong evidence that part of the variation in host preference within a vector species is attributable to vector genetics [31]. Among *Aedes* species, the forest form of *Aedes aegypti* is zoophilic, showing preference for animals, whereas the domestic form is anthropophilic [32]. McBride *et al*. [23] demonstrated the role of genetic variation in chemosensory genes underlying this behavioural difference. They showed that the evolution of human odour preference is linked with increased expression of the odorant receptor *Or4* [23]. Other candidate genes showing expression levels that correlated with host preference were also identified [23], suggesting that multiple genes play a role in the evolution of this complex behaviour. In *An. arabiensis*, genomic inversions have been found to be associated with the host species that specimens have fed on, suggesting that genetic differences in these genomic regions affect vector host preference [33]. Transcriptomic analysis has since been used to compare the highly anthropophilic species *An. coluzzii* with the zoophilic *Anopheles quadriannulatus*, and suggested that differences in chemosensory genes underlie variation in host preference [34]. Since most studies have only shown correlations between feeding behaviour and variation in the chemosensory genes, future work should aim to validate the function of these genes, in particular through genetic engineering [23,35]. The recent development of gene-editing tools such as CRISPR–Cas9 in mosquitoes has enhanced the ability to understand the molecular basis of host detection [36]. These techniques have been used to disrupt the function of candidate olfactory genes in mosquitoes, allowing the assessment of the relative contribution of these genes to human odour detection and blood feeding [24].

### (b) Human host attractiveness: ‘smell me if you can’

The human body produces over 350 VOCs, of which very few are human-specific [37,38]. Many VOCs have been shown to elicit a behavioural or electroantennographic response in receptive mosquito species [14,39]. Mosquitoes show attraction to some VOCs, such as 3-methyl-1-butanol [40], and repellence by others, such as 6-methyl-5-hepten-2-one, octanal, nonanal, decanal and geranylacetone. Although VOCs have been shown to affect mosquito behaviour when tested individually, the interaction is likely to be far more complex. Mosquitoes also react to combinations of volatiles that may act in synergy or antagonize each other [41], and removal or addition of compounds can affect the attractiveness of the blend [42,43]. While mosquito host selection in nature depends on a variety of factors, anthropophilic mosquito species show strong preferences to human odour above other odours such as cattle in laboratory studies [44] and there are clear interspecific differences in volatile profiles [45]. This suggests that variation in VOCs may play a role in anthropophily; however, further research is required to pinpoint which VOCs contribute the most to host selection.

Behavioural assays and electrophysiological experiments have shown that mosquitoes, predominantly *Anopheles* and *Aedes*, can also distinguish between odours emanating from different people [15,46,47]. For example, people less attractive to *Ae. aegypti* tend to produce more of certain volatiles, including octanal, nonanal and decanal [14], suggesting that these may act as natural repellents. Other studies have reported variation in attractiveness to several *Aedes* and *Anopheles* mosquito species with factors such as pregnancy [48–50], diet [51], consumption of alcohol [52,53] or age [16,54]. The exact biological mechanisms behind these effects are largely unknown, and it is not clear to what extent they are mediated by changes in body odour. For example, adults have been found to attract *Anopheles* mosquitoes more than children [16], but age correlates with physiological changes including maturation of eccrine and sebaceous glands and an increase in body mass [55], therefore, disentangling the underlying mechanisms remains challenging. Similarly, pregnant women have been shown to be twice as attractive to *Anopheles* mosquitoes as non-pregnant women, which could be attributable to a variety of factors such as their higher body mass, increased body temperature or hormonal status [48]. More longitudinal studies, examining the same individuals over time to control for temporal variation, and larger sample sizes are needed to understand the complex mechanisms underlying variation in attractiveness.

While progress has been made in characterizing the VOCs that humans produce, and what role these may have in attractiveness to mosquitoes, the biosynthetic pathways leading to the production of human-derived volatiles remain elusive [37]. Nonetheless, recent findings suggest an important role of genetics in attraction. Early questionnaire-based studies suggested more concordance in attractiveness to mosquitoes between monozygotic (identical) than dizygotic (non-identical) twins [56]. However, while such surveys are cost-effective and allow for large sample sizes, their explanatory power is limited by potential response biases and other confounding effects. More direct support for the heritability of attractiveness has come from experimental studies with twins. Fernández-Grandon *et al.* [57] found a stronger correlation in attractiveness between monozygotic twins compared with dizygotes [57]. Interestingly, other twin studies also reported a genetic basis to human body odour [58,59]. Studies with mice have demonstrated how body odours are regulated by genes of the major histocompatibility complex (MHC) [60], but the mechanism by which MHC genes exert their influence has not been identified [61,62]. Attempts to identify a role of the MHC in attraction to mosquitoes have produced inconsistent results, with only evidence of a weak association being described thus far [63]. Further evidence for the role of genetics in attractiveness may come from studies making use of genome-wide data collected on large cohorts of twins such as the one being conducted in our group where the aim is to link genetic data to attractiveness to mosquitoes, the production of VOCs, and other factors such as the skin microbiome.

## 3. Microbes: the ‘dark matter’ of mosquito–human interactions

Humans and mosquitoes interact with microbes in their environment and this can profoundly affect their phenotype, including traits involved in disease transmission such as immunity [64,65]. There is growing evidence that microbes can also affect the human odour, altering the host's level of attractiveness and ultimately transmission of disease (figure 1).

### (a) Skin microbiome: the essence of scent?

Bacteria living on the human skin are thought to be significant producers of VOCs [66]. Specifically, skin bacteria are known to catabolize and convert skin lipids and aliphatic amino acids, present in sebum and sweat, respectively, into short-chain carboxylic acids [67]. Early studies showed that freshly secreted sweat is odourless, but incubation with bacteria leads to a characteristic smell [68]. Freshly secreted sweat is only minorly attractive to *An. gambiae* mosquitoes, whereas incubated sweat is more attractive [69], and bacteria cultured on artificial medium have been shown to produce VOCs that attract mosquitoes [70]. Since the presence of skin bacteria can influence mosquito feeding behaviour, it can be hypothesized that differences in skin microbiome composition among people may lead to differences in attractiveness to mosquitoes. Indeed, individuals that are more attractive to mosquitoes were found to harbour a higher abundance but a lower diversity of bacteria on their skin compared with less attractive people [71]. Given the high species diversity of the human skin microbiome, further work should explore the contribution of individual bacterial taxa to levels of attractiveness. Alternatively, the sum of these individual effects or more complex ‘cocktail effects’ may be at play. It has been shown that the human skin microbiome is partly heritable, with Gram-negative bacteria of *Roseomonas* genus being found as the most heritable in a study of Korean twins [72]. Another interesting result from this study was that the abundance of *Corynebacteria* was found to be associated with a polymorphism in a gene related to epidermal barrier function [72]. Verhulst *et al.* [73] had previously found *Corynebacteria* to produce volatiles that attract mosquitoes, which suggests that the effect of skin bacteria on attractiveness could be controlled by host genetic factors involved in regulating the skin micro-environment. The human skin also harbours fungi that produce VOCs [74], but the role of these compounds in mosquito–human interactions is largely unexplored. In addition, viruses and archaea are present on the skin [75], and may also contribute to mosquito attraction. To date, most studies of the skin microbiome in relation to attractiveness have relied on amplicon sequencing of the bacterial 16S gene, which mostly provides good resolution at the genus level [71]. This is likely to change in the near future with major technological advances being made in shotgun metagenomic sequencing providing multi-kingdom and strain-level resolution [76,77].

Given some evidence that different food regimes can alter the skin microbiome [78], it is possible that diet may indirectly affect attractiveness to mosquitoes through changes in bacterial communities on the human skin. Some human genes could also promote the production of particular compounds on the skin that allow specific bacteria to grow, but heritability in the skin microbiome may also be explained by the fact that bacteria are transmitted from mother to offspring at birth [79]. Current knowledge on the topic suggests that this effect might be transient as the skin microbiome composition undergoes profound alterations as children become adults [80]. It appears likely that both genetic and environmental factors affect the composition of the skin microbiome [72] and that interactions between these effects, coupled with the VOCs produced by the body, contribute to levels of attraction to mosquitoes.

Interestingly, there are differences in skin microbiome between humans, other primates and cattle, in terms of both diversity and composition [45,81], and this could potentially influence mosquito preference for particular host species. For instance, although less diverse, the human skin microbiome was found to have a higher abundance of *Staphylococcus* spp., known to be attractive to *Anopheles* mosquitoes, compared with other apes and monkeys [45,71].

Microbial communities also reside in other body sites, including the gut. The gut microbiota impacts metabolic functions and immune responses in the body, and their composition is thought to be important in human health [82]. Previous studies have demonstrated links between body odour and disease [83–85], while recent research demonstrated a link between volatiles in the breath and the gut microbiota [86]. It would, therefore, be of interest to investigate whether other microbial communities in the body, particularly the gut, correlate with VOC production and how parasite infection may affect this.

## 4. Parasites: puppet masters of mosquito–host interactions?

Parasites often alter their host's phenotype beyond the mere pathological effects of the infection by inducing various physiological or behavioural changes. In some cases, the occurrence of these changes is the product of evolution acting on the parasite's genome and selecting for parasites that can ‘manipulate’ the host phenotype in ways that increase parasite transmission [87].

### (a) Infection-associated changes in mosquito behaviour

*Plasmodium*-infected mosquitoes have been shown to be more attracted to blood hosts, more persistent at biting and to feed more frequently when they carry the infective (sporozoite) stage of the parasite, while their motivation to feed on blood was shown to decrease when infected with the earlier stage of the parasite [88,89]. By contrast, other studies have reported no change in behaviour following infection of mosquitoes [90]. The results from different studies are difficult to compare as they vary in the model system used, experimental design and methods. In particular, discrepancies may result from host–parasite coevolution. For example, differences in mosquito genotype may impact the interaction of the mosquito with a particular strain of parasite. Most studies have used non-sympatric vector–host–parasite combinations which may fail to reveal the effects on mosquito behaviour that may have developed in sympatric species. However, even the use of parasites and vectors isolated from the same location in one study found no evidence of *Plasmodium*-induced behaviour [90], suggesting that there is variation in the induction of the behavioural change. This theory is supported by Stanczyk *et al*. [91], where different *Anopheles–Plasmodium* combinations caused species-specific alterations in mosquito olfactory responses. More studies using sets of sympatric (coevolved) host–parasite combinations should be conducted to understand this variation.

To date, both the extent of behavioural manipulation and the underlying mechanisms remain unclear [92]. While we do not know if malaria parasites can directly alter behaviour, there is evidence that this effect could be indirectly mediated by the mosquito response to the infection. Indeed, the effect of *Plasmodium* on mosquito behaviour can be replicated by an immune challenge with the bacterium *Escherichia coli* [93], suggesting this effect is not specific to malaria parasites, and other factors such as insect immunity might be involved. Nevertheless, these changes would still be expected to increase the mosquito–host contact rate and therefore the transmission of malaria parasites [94]. While immune-challenged mosquitoes showed changes in antennal responses to certain compounds, supporting the hypothesis of a general effect of infections, species-specific alterations of mosquito olfaction have also been demonstrated [91]. This implies that at least some changes in antennal responses to odours are malaria-specific, and can vary depending on both the mosquito species and *Plasmodium* species involved. In some cases, changes in behaviour may even shift the vector's host preference towards the host species that is the most suitable to the parasite's survival, as suggested in a study showing increased anthropophagy in mosquitoes infected with sporozoites of the human malaria parasite *Plasmodium falciparum* [95]. Interestingly, some mosquito-borne viruses such as dengue or Lacrosse virus have also been shown to modify the behaviour of several *Aedes* species in ways that could enhance their transmission, suggesting that manipulation of feeding behaviour might also be common beyond malaria systems [96,97]. Further research is needed to fully understand the effect of *Plasmodium* and virus infections on mosquito behaviour and assess to what extent these may benefit their transmission [98].

### (b) Infection-associated changes in host attractiveness

Several studies have now demonstrated that mammalian hosts, including humans, become more attractive to mosquitoes when infected with infective *Plasmodium* gametocytes [99–101]. There is growing evidence this effect is mediated by changes in body odour of infected people [101,102], with particular volatiles commonly found in uninfected people being at either lower or higher concentration in malaria-infected people. It is possible that these derive from the human host, its skin microbiome or the parasites themselves [101,102].

There is evidence that metabolites directly produced by the *Plasmodium* parasite could be driving the change in body odour. In red blood cell lines, *P. falciparum* secretes a metabolite, (*E*)-4-hydroxy-3-methyl-but-2-enyl pyrophosphate (HMBPP), that triggers an increase in the production of CO_2_, aldehydes and monoterpenes by the infected cells. Importantly, this induced response of blood cells was found to enhance vector attraction and feeding on the infected blood [103]. While this observation is interesting, it remains to be tested whether HMBPP induces changes in VOCs on the human skin and if its effect on attractiveness can be replicated in a living host. Interestingly, increased production of some of the same aldehydes was also found by Robinson *et al.* [101], providing some support for this mechanism. Alternatively, or additionally, aldehydes are oxygenated compounds that can be synthesized during lipid peroxidation caused by oxidative stress. Malaria-induced oxidative stress is a known phenomenon [104] and may be another potential mechanism to explain the increased production of aldehydes.

Therefore, it remains to be established to what extent changes in infected hosts may be malaria-specific as well as whether they are mediated directly by the parasite or indirectly through changes in host metabolism or immune status in response to the infection. In support of the latter hypothesis, body odour is known to contain chemosensory cues associated with diverse infections and illnesses [83–85]. However, in field study populations such as those used in Robinson *et al*. [101], participants were likely to harbour other infectious organisms such as helminths, which can also lead to oxidative stress [105]. This suggests that if the change in the body odour chemical signature was due to a general infection, there would have been no difference between individuals who were malaria-free, but had other infections, and *Plasmodium*-infected participants, which was not observed. Additionally, participants were asymptomatic and their odour profile returned to normal after antimalarials were given. Future studies should aim to identify the underlying cause and confirm whether the identified odour signature associated with *Plasmodium* infection is malaria-specific or is due to oxidative stress caused by a general infection. A comparison of odour samples from single and co-infections (e.g. with helminths) before and after antimalarial treatment would be highly valuable for answering this question.

Nevertheless, there is also some evidence that changes in body odour are partly controlled by malaria parasite genomes. Indeed, one study found differences in the skin odour profile of participants infected with two different strains of *P. falciparum*, suggesting that parasites can vary in their effect on the host body chemistry [102]. Further research should try to replicate these results by comparing different parasite strains across different host genetic backgrounds, developmental stages or immunological states. In particular, the parasite density can vary between individuals and it has been demonstrated that children with microscopic densities of gametocytes show an increase in attractiveness, whereas children with submicroscopic densities do not [100]. Parasitaemia also varies between children and adults as well as between symptomatic and asymptomatic individuals, potentially owing to differences in levels of acquired immunity [106,107]. However, it is not known if the effect of *Plasmodium* on attractiveness varies between these groups. Understanding how parasite infection can affect host attractiveness has important implications not only for transmission ecology and modelling, but also for control methods, including improved odour-based traps, or novel traps specifically targeting malaria-infected mosquitoes and removing these from the population.

## 5. Evolutionary ecology of vector biting, a missing piece in the puzzle?

Although differences in feeding behaviour between mosquito species and variation in attractiveness between host species have been known for a long time, individual variation within each species has often been considered as source of statistical noise rather than a biologically relevant feature of the interaction [46] However, both genetic and non-genetic factors underlying this variation could significantly affect the outcome of the mosquito–human interaction and, therefore, the dynamics of disease transmission in the field. Moreover, studies have usually considered one source of variation in isolation, so it is unclear how the combination of different sources of variation may impact the outcome of the interaction.

**Figure 2.**
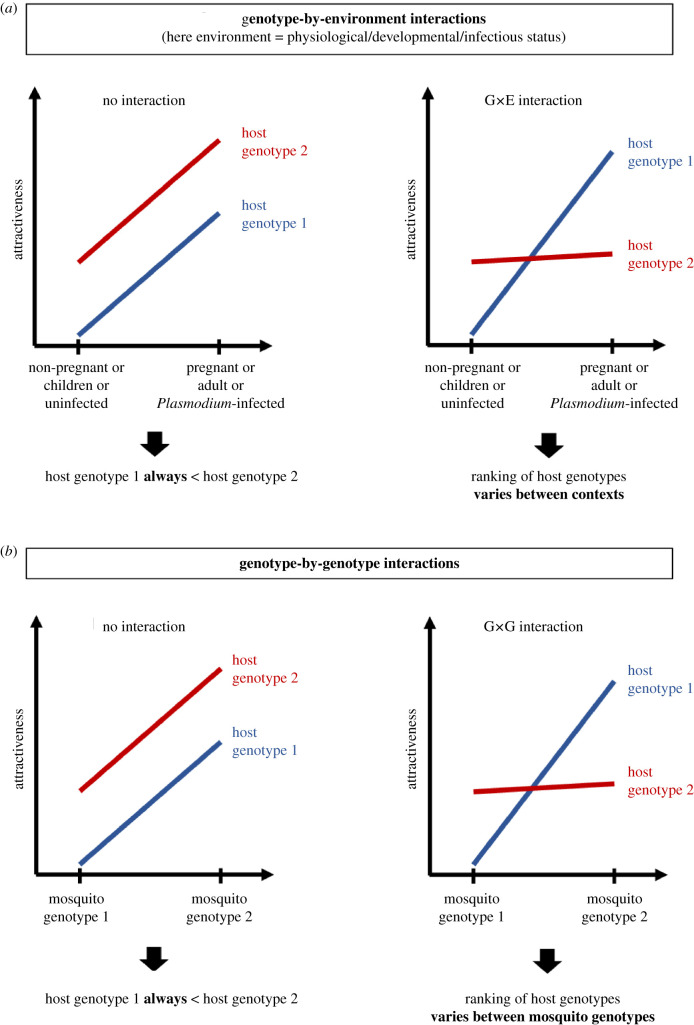
Potential role of interactions between genetic and environmental factors. (*a*) Where there is no G × E interaction, a given host genotype is expected to remain less attractive than another host genotype independent of *Plasmodium* infection, pregnancy or development stage. If there is G × E interaction, the relative attraction may be reversed. (*b*) G × G interaction could also lead to changes in relative attraction depending on the genotypes of the host and mosquito. (Online version in colour.)

For instance, it is not known if the magnitude of the effect of pregnancy on attractiveness to mosquitoes varies with the female host's genetic background. Similarly, the host genotype could interact in a complex manner with the effect of age or *Plasmodium* infection described above, meaning that the ranking of individuals regarding their attractiveness varies depending on their developmental or infection status. Such relationships between genetics and pregnancy, developmental or infection status can be interpreted as a form of genotype-by-environment (G × E) interaction, where the environment is any non-genetic factor that affects the phenotypic expression in a given genotype (figure 2*a*).

Assuming that there are genetic factors underlying variation in both the human host and the mosquito vector, levels of attractiveness may also depend on the nature of the genotype-by-genotype (G × G) interaction between them (figure 2*b*). For example, while it is known that some individuals are less attractive hosts [14,15], the levels of attractiveness could depend on the particular mosquito genotype tested, thus making the term ‘less attractive’ misleading if these individuals encounter mosquitoes with different feeding preferences in the field. This hypothesis prompts further research involving genotypes of hosts and vectors that are locally adapted to each other. Interestingly, Logan *et al*. [41] tested the repellency effect of human-derived odour blends against three species of mosquitoes and found similar results across species, suggesting that some people may produce wide-spectrum natural repellents. More complex outcomes could emerge if more sources of variation are added to the equation, such as genotype-by-genotype-by-environment interactions (G × G × E) or interaction with genetic variation in the parasite. Moreover, these interactions may also be relevant at the inter-species level, for instance when several species of mosquitoes, hosts and parasites coexist in the same environment [95,108,109]. Therefore, it is crucial to assess the relative contribution of each potential factor to the overall variation in attractiveness in order to identify the ones that are the most biologically important.

The potential role of genetic variation raises interesting questions about the action of natural selection and coevolution between hosts, vectors and parasites. On the one hand, levels of attractiveness in human populations exposed to disease-transmitting vectors are likely to be under strong selective pressure. Provided that this trait is heritable and that individuals who are poorly attractive to mosquitoes are less likely to become infected, this could lead to changes in levels of attractiveness over generations and potentially genetic differentiation between populations with different risk of contracting a mosquito-borne infection. There is considerable heterogeneity in malaria transmission in endemic areas, with a minority of individuals receiving the majority of infections [110], and a recent study has shown that there is also considerable variation in biting frequency of *Anopheles* mosquitoes, with a small proportion of the population receiving the majority of the bites [111]. This suggests there is significant variation in malaria transmission potential within a population, and prompts further research to test whether it can be explained by genetic differences in attractiveness between people.

Profiling of the human leukocyte antigen (HLA) genes of the MHC, which are considered to be involved in the regulation of human body odour, suggests that people carrying the HLA gene Cw^∗^07 are more attractive to mosquitoes [63]. While the evidence for a positive correlation between carrying Cw^∗^07 and high attractiveness of human skin emanations is relatively weak and would need further validation, it is interesting to note that the frequency of Cw*07 in malaria-endemic countries is significantly lower than in other regions, which could be indicative of human population adaptations in response to the selective pressure of vector-borne diseases (VBDs) [112]. A possible link between HLA genes and attractiveness to mosquitoes raises important questions in the context of immunity to malaria. HLA genes are thought to be involved in controlling the parasite infection [113] and it would be interesting to test whether levels of attractiveness could be positively or negatively correlated to immunity, as these two scenarios could have very different outcomes on disease epidemiology.

If natural selection acting on levels of attractiveness is confirmed, we could expect selection for mosquito genotypes with altered host preferences as the frequency of highly attractive hosts decreases, potentially leading to a coevolutionary arms race between host attractiveness and mosquito feeding behaviour. Although evidence of adaptive shifts in mosquito preference towards certain genotypes of the same host species is still lacking, drastic changes in human population density across Africa has likely facilitated the specialization of *Ae. aegypti* in biting humans over other animals [114].

## 6. Taking advantage of individual variation for disease control

The influence of genetic and non-genetic factors on mosquito behaviour, host attractiveness and parasite-induced changes may lead to strong heterogeneities in the effective contact rate between the three partners. Characterizing this variation and assessing the contribution of each factor should bring a more comprehensive view of disease epidemiology and, therefore, improve the way we study, predict and control the spread of VBDs.

### (a) Towards more realistic epidemiological models

Epidemiological models have generally considered the contact rate between vectors and hosts as a population average rather than an individual-specific variable. However, predictions can be very sensitive to variation in this parameter [12]. For instance, several studies have shown that integrating such heterogeneities provides better estimates of key epidemiological parameters such as the (*R*0) [12,16]. The existence of super-spreaders, i.e. individuals that contribute more to the parasite transmission (either because they are more infectious or because they attract more vectors), has been shown to govern inter-individual transmission dynamics for many infectious diseases, often with a small percentage of individuals contributing to the majority of transmission events [115]. This is important to consider since models that include the presence of super-spreaders provide very different outcomes in terms of disease extinction and outbreaks compared with average-based approaches [116]. Furthermore, understanding the role that infection plays in the host–vector interaction is also important for more realistic models, as parasite-associated changes in behaviour or host attractiveness are likely to significantly affect transmission ecology [91,95]. Characterizing heterogeneities in the human infectious reservoir, or even in vector populations, would also give more realistic expectations on the outcome of disease control interventions. For example, ignoring heterogeneity in exposure to infectious bites leads to underestimates of the efficacy of potential vaccines [117].

While the host's immune response is a major determinant of parasite epidemiology [118], similarities in odour profile or a lack of host specificity may allow for the transmission of parasites between two species [45,119], and indeed there is evidence that both *P. falciparum* and *Plasmodium vivax*, major pathogens in humans, evolved from parasites that infected African apes [120]. Host selection, therefore, has important implications for the epidemiology of disease, and understanding why mosquitoes show a preference could help to predict and prevent future outbreaks from sylvatic transmission cycles.

It remains to be seen to what extent the sources of individual variation reviewed above affect disease transmission in the field. Are very attractive people super-malaria spreaders? Are infected people concentrating most of the bites because of their increased attractiveness? Does the increase in the frequency of multiple biting by infected mosquitoes lead to more infectious bites than expected? A recent epidemiological model predicted that *Plasmodium*-induced behavioural changes in the number of lifetime bites could cause a doubling in the force of infection [93]. In addition to changes in biting rates, potential parasite-induced changes in the vector's preference for humans over alternative hosts has been predicted to lead to more than 250% increase in the parasite's transmission potential [95]. Such predictions highlight the need for modelling approaches that explicitly integrate the biological complexity of vector–host–parasite interactions.

### (b) Innovative control strategies, how can we fight back?

In the future we may be able to apply a targeted approach, searching for malaria-super-spreaders and focusing treatment on these individuals instead of trying to reach 100% coverage with LLINs and IRS. Where there is residual transmission, VBDs like malaria will continue to pose a threat because of outdoor biting despite universal LLIN and IRS coverage being achieved [121]. Heterogeneities in transmission are due to the fact that a high proportion of bites are carried by a small proportion of the population [115], which gives further evidence that attractive hosts are a worthy target. Indeed, targeting those individuals that contribute the most to parasite transmission is predicted to outperform population-wide measures in reducing the force of infection [12]. Identifying such individuals could be achieved using non-invasive techniques to detect specific VOCs associated with higher levels of attractiveness to mosquitoes or with the infection in asymptomatic individuals. Recent developments that have allowed for detection dogs to non-invasively and rapidly identify malaria-infected people [122] suggest that dogs could be trained and deployed in the field to recognize super-spreaders, or those that are highly attractive to mosquitoes and consequently more at risk of becoming infected. Further research into the odour profiles could also lead to the development of odour sensors as a simple non-invasive diagnostic tool for asymptomatic infection. Through such initiatives, resources for treatment and protection could be focused on these ‘super-spreaders’ to have community-wide reduction in the spread of VBDs.

Investigating naturally occurring blends of VOCs could also lead to the development of novel topical repellents that can mask body odour, reducing bites and therefore transmission. More advanced novel products could allow the manipulation of the human body odour or skin microbiome to reduce the production of attractive VOCs, therefore, reducing a person's attractiveness to mosquitoes. For instance, understanding genetic associations with attractiveness could lead to the development of drugs that target proteins controlled by the genes associated with attractive phenotypes. Furthermore, creating new blends of VOCs more similar to human odour could improve trapping methods for mosquitoes. This may include the addition of the aldehydes identified by Robinson *et al*. [101] to current synthetic attractants mimicking human body odour to divert mosquitoes away from infected individuals, and potentially create enough selection pressure to result in mosquitoes no longer responding to human odours. Other traps could be developed with lures that specifically target malaria-infected mosquitoes. The development of such tools will benefit from further research into the skin microbiome that reveals more VOCs produced by bacteria and fungi that affect mosquito feeding behaviour.

As mosquitoes are reliant on olfaction for host seeking, research into olfactory genes and the effect of *Plasmodium* infection will further our understanding of how mosquitoes locate a host and how infection can influence this. If appropriate olfactory genes are knocked out or altered, this could reduce the mosquito's ability to detect a host, or even shift their host preference away from humans, and could be used in future genetic control programmes.

## 7. Conclusion

Understanding why mosquitoes show variation in host preference, both between and within species, is highly important for future VBD control and understanding transmission. While it is generally accepted that these differences are mediated by variation in the volatile compounds produced by hosts [100], there is still much to discover about the mechanisms underlying the production of these compounds and how infection of the host or vector can affect either the production of or mosquito responses to VOCs. Mosquito behaviour in response to a variety of compounds has already been used to produce synthetic attractants such as MB5 [40] for use in traps and a variety of commercially available repellents such as DEET. However, a greater understanding of human attractiveness to mosquitoes and the effect of parasite infection could lead to improved control tools, including novel traps, repellents, drugs and gene drive programmes. This field of research is also likely to benefit from more studies integrating variation in attractiveness into an evolutionary framework, as this may help design of better control methods and predict the long-term consequences of field interventions.

## Data accessibility

This article has no additional data.
